# A comparative study of varying doses of enoxaparin for thromboprophylaxis in critically ill patients: a double-blinded, randomised controlled trial

**DOI:** 10.1186/cc12684

**Published:** 2013-04-19

**Authors:** Sian Robinson, Aleksander Zincuk, Ulla Lei Larsen, Claus Ekstrøm, Mads Nybo, Bjarne Rasmussen, Palle Toft

**Affiliations:** 1 Department of Anaesthesia and Intensive Care, Odense University Hospital (OUH), Sdr. Boulevard 29. Odense C, DK 5000, Denmark; 2 Department of Biostatistics, University of Southern Denmark, J.B. Winsløws Vej 9B, 2. Odense C, DK 5000, Denmark; 3 Department of Clinical Biochemistry and Pharmacology, OUH, Sdr. Boulevard 29. Odense C, DK 5000, Denmark

## Abstract

**Introduction:**

Critically ill patients are predisposed to venous thromboembolism. We hypothesized that higher doses of enoxaparin would improve thromboprophylaxis without increasing the risk of bleeding. Peak anti-factor Xa (anti-Xa) levels of 0.1 to 0.4 IU/ml reflect adequate thromboprophylaxis for general ward patients. Studies conducted in orthopaedic patients demonstrated a statistically significant relationship between anti-Xa levels and wound haematoma and thrombosis. Corresponding levels for critically ill patients may well be higher, but have never been validated in large studies.

**Methods:**

Eighty critically ill patients weighing 50 to 90 kilograms were randomised in a double-blinded study to receive subcutaneous (sc) enoxaparin: 40 mg once daily (QD), 30 mg twice daily (BID), 40 mg BID, or 1 mg/kg QD, each administered for three days. Anti-Xa activity was measured at baseline, and daily at 4, 12, 16 and 24 hours post administration. Antithrombin, fibrinogen, and platelets were measured at baseline and twice daily thereafter.

**Results:**

Two patients were transferred prior to participation. On day 1, doses of 40 mg QD (n = 20) and 40 mg BID (n = 19) yielded mean peak anti-Xa of 0.20 IU/ml and 0.17 IU/ml respectively. A dose of 30 mg BID (n = 20) resulted in much lower levels (0.08 IU/ml). Patients receiving 1 mg/kg QD (n = 19) achieved near steady-state mean peak anti-Xa levels from day 1 (0.34 IU/ml). At steady state (day 3), mean peak anti-Xa levels of 0.13 IU/ml and 0.15 IU/ml were achieved with doses of 40 mg QD and 30 mg BID respectively. This increased significantly to 0.33 IU/ml and 0.40 IU/ml for doses of 40 mg BID and 1 mg/kg QD respectively. Thus anti-Xa response profiles differed significantly over the three days between enoxaparin treatment groups (*P *<0.0001). Doses of 40 mg BID and1 mg/kg QD enoxaparin yielded target anti-Xa levels for over 80% of the study period. There were no adverse effects.

**Conclusions:**

Doses of 40 mg QD enoxaparin (Europe) or 30 mg BID (North America) yield levels of anti-Xa which may be inadequate for critically ill patients. A weight-based dose yielded the best anti-Xa levels without bioaccumulation, and allowed the establishment of near steady-state levels from the first day of enoxaparin administration.

**Trial registration:**

Current Controlled Trials ISRCTN91570009.

## Introduction

The critically ill patient is especially predisposed to venous thromboembolism (VTE), possessing many inherent risk factors: history of VTE, renal insufficiency, cardiac failure, trauma, sepsis, cancer, increasing age, and obesity [1-3]. The risk of VTE is approximately 1% per day amongst some subgroups of critically ill patients [2]. When compared with patients who did not have VTE, those with VTE are reported to have a longer duration of mechanical ventilation, longer duration of intensive care unit (ICU) or hospital stay, and higher hospital mortality [4].

Thromboprophylaxis with agents such as enoxaparin, a low- molecular-weight heparin (LMWH) is justified in most ICU patients [1,5,6]. The effect of subcutaneous (sc) enoxaparin on the coagulation cascade is thought to be reflected by anti-factor Xa (anti-Xa) levels [7], however, this surrogate marker for efficacy has never been validated in suitably sized studies. Normally, anti-Xa levels peak 3 to 5 hours after dosing [8]. For sc administration, steady-state levels are achieved after the third dose [9].

LMWH is often employed as a safe and effective means of prophylaxis against VTE in medical and surgical patients. Conventional target peak anti-Xa levels for thromboprophylaxis in this population are 0.1 to 0.40 IU/ml [10]. Corresponding levels for critically ill patients are unknown, but are likely to be higher. Levine's study conducted in orthopaedic patients, demonstrated a statistically significant relationship between anti-Xa levels and the occurrence of wound haematoma and thrombosis. Furthermore, regression analysis suggested anti-Xa levels were predictive of outcome [11].

Previous studies in critically ill patients demonstrated sub-therapeutic levels when the European standard daily dose of 40 mg enoxaparin was administered [12,13]. VTE also tended to occur more frequently during the ICU stay as compared to the subsequent period on the wards. Despite ICU patients receiving LMWH, 5.1% to 15.5% developed proximal leg deep vein thrombosis in studies conducted by Fraisse and Cook *et al*. [4,14]. This study aims to establish the optimal dose of enoxaparin for ICU patients. We hypothesised that higher doses of enoxaparin would optimise thromboprophylaxis without increasing the risk of bleeding in these patients.

## Materials and methods

### Study population

Sample population consisted of 78 consecutive eligible patients admitted to the ICU at Odense University Hospital in Denmark who were ≥18 years of age, with a minimum stay of >24 hours. Patients weighing <50 kg or >90 kg were excluded. Likewise, patients with coagulopathy (international normalised ratio (INR) >2 times upper limit of normal, or activated partial thromboplastin time (aPTT) >2 times the upper limit of normal), or severe thrombocytopenia (platelet count <75 × 109/L), pregnant patients, patients requiring therapeutic anticoagulation, or those with contraindications to heparin, limitation of life support or renal failure (creatinine clearance (CrCl) <30 ml/min/1.73 m²) were deemed ineligible. We recorded patient demographics, diagnosis on admission and standard ICU severity of illness scores on the day of entry into the study.

### Design

A prospective randomised double-blinded study was conducted on a mixed ICU. Patients were randomised to receive sc enoxaparin: 40 mg once daily (QD) (control group), versus 30 mg twice daily (BID), 40 mg BID or 1 mg/kg QD (test groups) each administered for 3 days (that is three doses total for QD regimens, and six doses total for BID regimens). Enoxaparin was administered to the thigh or abdomen of the study patients. Anti-Xa activity was measured at baseline, and at 4, 12, 16 and 24 hours post administration of enoxaparin on each day during the study period. Antithrombin (AT), fibrinogen, and platelets were measured at the baseline, and thereafter twice daily with the average on each day being recorded. Patients, family members, clinicians, and research personnel, were all unaware of study-group assignments. Nurses, who administered the drug, were the only party privy to the actual dose given to each patient as it was impossible to prepare a dose of 1 mg/kg beforehand. A fall in CrCl to <30 ml/min/1.73 m² during the 3-day study period would result in the patient being counted as a dropout.

The study was approved by the science ethics committee of Vejle and Funen. It was performed in accordance with the ethical principles set forth in the Declaration of Helsinki, and with local regulations, and monitored by the committee for Good Clinical Practice (GCP). Written informed consent was obtained from each patient where possible or otherwise from the closest family member prior to enrolment in the study.

Funding sources for this research had no role in the design, data collection, analysis, interpretation or reporting of this study.

### Assay methods

Samples for anti-Xa activity were analysed using a validated chromogenic assay kit (COAMATIC™ Heparin, Chromogenix, Instrumentation Laboratory Company, Lexington, MA, USA). A LMWH curve was utilised.

Furthermore, the anti-Xa analysis was calibrated towards the specific LMWH type to avoid differences that have been reported when using a standard calibration. AT and fibrinogen were analysed using STA-R instrument (STAGO, Triolab A/S, Brøndby, Denmark) with dedicated reagents used according to the manufacturer's instructions. Platelet counts were performed on a Beckman Coulter LH750 (Beckman Coulter, Brea, CA, USA).

### Outcome measures

Primary endpoint was to determine the optimal enoxaparin dose. The optimal enoxaparin dose was defined as that dose which would yield the highest target anti-Xa levels, allow patients to remain within target anti-Xa levels for >75% of the study period, and would not cause an increase in major bleeding events. Secondary endpoints included the identification of any correlation between the dose of enoxaparin and (i) levels of AT, fibrinogen, platelets, and (ii) bleeding - major and minor.

### Statistical methods

The null hypothesis was that there was no difference in peak anti-Xa levels between the groups. On the basis of data from previous studies, this study was designed with 80% power to detect a 30% absolute difference in anti-Xa levels between the groups, giving a total sample size of 80 patients. All baseline demographic values were compared using one-way ANOVA (numerical data) or chi-squared test (categorical data). Statements of statistical significance are based on a two-tailed test with a level of 0.05. Individual change over time is pursued by repeated effects modelling, which allows for correlation among measurements taken on the same patient over time. Our initial model used APACHE II, SOFA, creatinine clearance, weight, age, gender, time, treatment allocation and an interaction between treatment and time as possible predictors for the anti-Xa levels. Subsequently, model simplifications were based on likelihood ratio tests. Patients were analysed in the group to which they were randomised, according to the intention-to-treat principle.

## Results

### Study population

**Figure 1:**
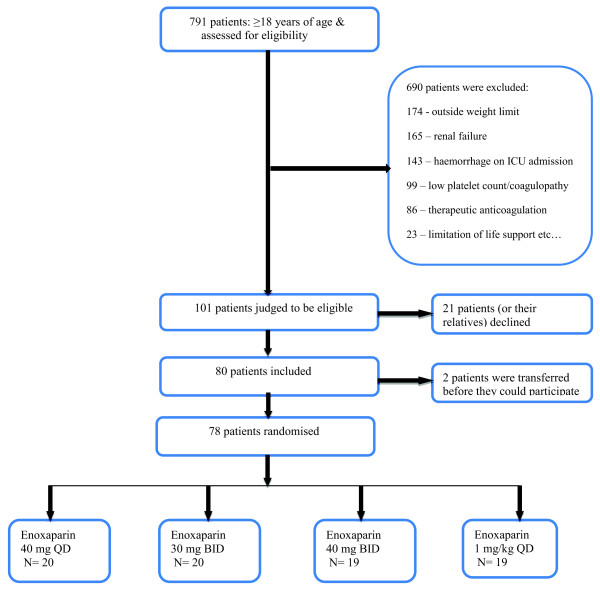
**Consort diagram**.

A total of 78 ICU patients were enrolled in the study between January 2011 and September 2012. The consent rate for participation was approximately 80%. The Consort diagram [15] shows patient disposition (Figure 1). During this period, we excluded 690 patients and randomised 80 patients. The most frequent reasons for exclusion were: 1) extremes of weight (25%); 2) creatinine clearance <30 ml/min/1.73m2 (24%); 3) haemorrhage on ICU admission (21%); 4) thrombocytopenia or coagulopathy (14%); 5) therapeutic anticoagulation (12%).

The majority of patients were admitted with respiratory failure due to pneumonia or exacerbation of chronic obstructive pulmonary disease (COPD) (73%). Other reasons for admission included: sepsis, intra-abdominal abscess, pancreatitis, and multiple trauma. At baseline, patients did not differ significantly between the control group (40 mg QD), and the three test groups. Patients had normal renal function and were moderately ill as assessed by severity of illness scores (Table 1).

**Table 1:** Clinical characteristics of the study population.

Enoxaparin dose	40 mg QD (N = 20)	30 mg BID (N = 20)	40 mg BID (N = 19)	1 mg/kg QD (N = 19)	*P *value
Sex (male:female)	12:8	14:6	8:11	9:10	0.31
Age (years)	59.9 ± 10.8	65.8 ± 14.7	65.6 ± 15.8	63.5 ± 13.8	0.44
Weight (kg)	69.6 ± 12.2	68.5 ± 10.9	67.8 ± 11.8	74.8 ± 9.4	0.71
SAPS II	40.2 ± 11.8	41.6 ± 10.4	44.9 ± 14.8	45.2 ± 16.6	0.18
APACHE II	20.7 ± 6.5	21.5 ± 6.0	22.6 ± 6.1	23.7 ± 7.4	0.80
SOFA	4.7 ± 1.7	4.6 ± 1.8	4.7 ± 1.4	4.4 ± 1.5	0.73
Creatinine clearance (ml/min)	83 ± 14.2	83 ± 12.8	80 ± 18.0	77.8 ± 18.4	0.33
Medical:surgical	17:3	19:1	16:3	17:2	0.73

Plus-minus values are means ± standard deviation (SD). QD, once daily: BID, twice daily; N, number; SAPS II, Simplified Acute Physiology Score II: APACHE II, Acute Physiology and Chronic Health Evaluation; SOFA, Sequential Organ Failure Assessment score.

### Primary results

**Figure 2:**
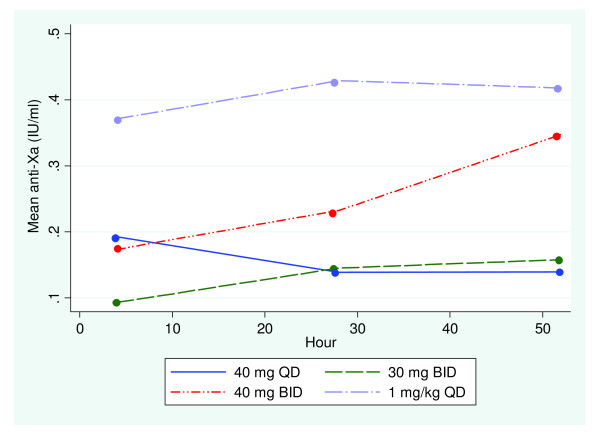
**Mean peak anti-factor Xa over time with varying doses of enoxaparin**.

**Figure 3:**
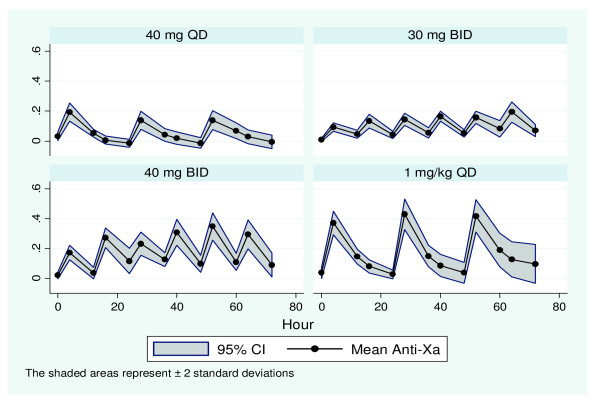
**Variation in anti-factor Xa over time for each dose of enoxaparin**.

Unblinding occurred on 1 October 2012. For patients with 1 mg/kg QD the mean administered dose of enoxaparin was 75 mg. Repeated measures modelling revealed that anti-Xa levels differed in a linear fashion with the dose of enoxaparin administered, and the time at which the blood samples were drawn. On the first day of enoxaparin administration, doses of 40 mg QD and 40 mg BID yielded similar mean peak anti-Xa of 0.20 IU/ml and 0.17 IU/ml respectively, while a dose of 30 mg BID resulted in much lower levels of anti-Xa (0.08 IU/ml). Patients receiving 1 mg/kg QD enoxaparin achieved near steady-state levels from day 1 of administration with mean peak anti-Xa levels of 0.34 IU/ml (Figure 2). Steady-state anti-Xa was achieved for all doses of enoxaparin at day 3. At steady state, mean peak anti-Xa levels of 0.13 IU/ml and 0.15 IU/ml were achieved with doses of 40 mg QD and 30 mg BID respectively. This increased significantly to 0.33 IU/ml and 0.40 IU/ml for doses of 40 mg BID and 1 mg/kg QD enoxaparin respectively. The anti-Xa response profiles were thus significantly different over the 3 days between the four treatment allocation groups (*P *<0.0001). Patients maintained anti-Xa levels within a target range of 0.1 to 0.4 IU/ml for: 33.3% (40 mg QD), 41.7% (30 mg BID), 83% (1 mg/kg QD) and 91.7% (40 mg BID) of the study period (Figure 3). There were no adverse effects.

### Secondary results

There was no significant difference in the AT, platelet count, or fibrinogen between the four groups (Table 2). There was no significant change from baseline in these coagulation parameters within each group over the 72-hour study period (Table 3).

**Table 2:** Coagulation parameters at baseline.

Parameter	Enoxaparin 40 mg QD	Enoxaparin 30 mg BID	Enoxaparin 40 mg BID	Enoxaparin 1 mg/kg QD	*P *value
Platelets (×10^9^/L)	285 ± 107	238 ± 112	223 ± 95	276 ± 121	0.78
AT (%)	87 ± 24	75 ± 20	89 ± 23	91 ± 23	0.90
Fibrinogen (mg/dL)	588.4 ± 194	527.2 ± 167	612.2 ± 180	547.6 ± 150	0.74

Data are means ± standard deviation (SD). QD, once daily; BID, twice daily; AT, antithrombin.

**Table 3:** Coagulation parameters at 72 hours.

Parameter	Enoxaparin 40 mg QD	Enoxaparin 30 mg BID	Enoxaparin 40 mg BID	Enoxaparin 1 mg/kg QD	*P *value
Platelets (×10^9^/L)	298 ± 108	299 ± 155	258 ± 129	276 ± 132	0.62
AT (%)	88 ± 35	85 ± 17	85 ± 33	87 ± 25	0.07
Fibrinogen (mg/dL)	571.4 ± 146	506.8 ± 180	612.2 ± 207	517 ± 170	0.69

Data are means ± standard deviation (SD). QD, once daily; BID, twice daily; AT, antithrombin.

### Adverse events

Four patients died during the course of the study - one patient from each group. These deaths were not a direct complication of enoxaparin use. All patients who died did so after withdrawal of active therapy. They had the following diagnoses: pneumocystis pneumonia, interstitial lung disease, lung metastases complicated by pneumonia and severe sepsis due intra-abdominal infection.

There were no episodes of bleeding. One patient experienced a fall in platelet count to half the baseline value within 2 days of starting the trial on 40 mg enoxaparin QD sc. However, this patient was also receiving azathioprine due to idiopathic lung fibrosis. There were no cases of heparin-induced thrombocytopenia.

## Discussion

In the present study, enoxaparin 1 mg/kg QD resulted in the highest target anti-Xa levels, and allowed patients to remain within target anti-Xa levels for >80% of the study period. There was no evidence of bioaccumulation or bleeding episodes.

Critically ill patients are predisposed to VTE. The 9^th ^American College of Chest Physicians' guidelines and the latest edition of Surviving Sepsis campaign guidelines recommend the use of LMWH or unfractionated heparin, to combat VTE in critically ill patients. Mechanical thromboprophylaxis with intermittent compression devices or graduated compression stockings are recommended in patients with a high bleeding risk or contraindications to heparin [6,16].

Anti-Xa levels are used to judge the degree of thromboprophylaxis. Among orthopaedic patients, one study was able to demonstrate that anti-Xa levels were predictive of outcome[11]. This surrogate marker for efficacy has never been validated in suitably sized studies, and the optimal levels for critically ill patients are not known.

In ICUs across Europe, a standard dose of 40 mg QD enoxaparin sc is often employed for thromboprophylaxis [17]. A dose of 30 mg BID sc enoxaparin is thought to be equipotent, and is used widely throughout North America. Riha *et al*. prospectively followed 63 surgical patients receiving a dose of either 30 mg BID or 40 mg QD enoxaparin. They found that a dose of 40 mg once daily resulted in significantly higher peak anti-Xa levels and a correspondingly lower incidence of VTE compared with 30 mg twice daily [18].

Our study showed that while a dose of 30 mg BID resulted in lower levels of anti-Xa on the first day of administration, at steady state this dose yielded mean anti-Xa levels that were nearly indistinguishable from that of 40 mg QD enoxaparin. The discrepancy in these findings may be due to the diverse patient population on our ICU which houses both medical and surgical patients.

Patients receiving 1 mg/kg QD enoxaparin achieved near steady-state anti-Xa levels from day 1 of administration, and this could be advantageous in a population where the occurrence of VTE can have grave consequences. Peak anti-Xa levels did not surpass maximum acceptable anti-Xa levels of 0.4 IU/ml for doses of 40 mg BID and 1 mg/kg QD enoxaparin. Higher anti-Xa levels are attractive in critically ill patients as they may confer better thromboprophylaxis. With an earlier study, we established that patients were more likely to maintain anti-Xa levels within the target therapeutic range for longer periods of time when given higher doses of enoxaparin [13]. Similar results were obtained with the present study. Here, doses of 1 mg/kg QD and 40 mg BID yielded target range anti-Xa levels for over 80% of the study period. However the latter dose resulted in target range anti-Xa levels even at trough measurements, and this accounts for the higher percentage yield. This may also give rise to bioaccumulation.

ICU patients have an apparent heparin resistance thought to be due to physiological mechanisms, or use of pharmacological agents for example vasopressors (may impair absorption of sc LMWH through adrenergic-mediated vasoconstriction of peripheral blood vessels), or the presence of multiple organ dysfunction (may alter drug metabolism, distribution, and binding to albumin and acute phase proteins) [10,19,20]. Thus other possible explanations for the difference in anti-Xa levels could be: differences in volumes of distribution or levels of hypoproteinemia or dose of vasopressor, as well as the presence/absence of sc oedema. Renal function and antithrombin levels were equivalent in all four groups and so are not thought to have contributed.

No significant change from baseline occurred in AT, platelet count, and fibrinogen, irrespective of the dose of enoxaparin. These findings are consistent with other studies, which found no change in these haemostatic parameters with LMWH use [12,13,19].

One patient experienced thrombocytopenia due to concomitant azathioprine administration. Thrombocytopenia in ICU patients may occur because of drug administration, use of dialysis, increased platelet consumption, or decreased platelet production. Thrombocytopenia in the critically ill may also be a marker of severe physiological stress [21].

No bleeding episodes occurred with the present study. This was not surprising, given the mean CrCl of 80 ml/min, the exclusion of patients with coagulopathy and the relatively short study period. Major bleeding occurred in 5.5% of ICU patients assigned to receive thromboprophylaxis with LMWH in a recent study conducted by Cook *et al*. [4]. Many critically ill patients experience bleeding episodes, the majority of which will be minor. Major or fatal bleeding in the ICU is thought to be associated with prolonged global tests of coagulation and low platelet counts rather than with the use of prophylactic anticoagulants or antiplatelet agents [22]. Such patients were excluded from the present study.

Periodic monitoring of anti-Xa levels is recommended in special populations, for example, pregnant patients, children, patients with acute kidney injury (AKI), or those at extremes of body weight [23].

With only 3 days of observation, our study was not designed to detect VTE - a clear limitation. Other limitations include single-centre experience, and not recording the presence/absence of oedema. The study was also confined to patients who weighed between 50 and 90 kg to allow for comparison with our earlier study. The use of concomitant vasopressor was not recorded, and may have influenced the results. Dörffler-Melly's study [20] from 2002 showed impaired absorption of sc LMWH through adrenergic-mediated vasoconstriction, however, in a later study conducted by Priglinger [12] in 2003, no such correlation was found between the anti-Xa activity and the dose of norepinephrine.

Strengths of the present study include daily screening for consecutive eligible patients thereby minimising the potential for selection bias. Also the validity of our results is ensured as the dependent variable (anti-Xa level) was not subject to interpretation bias. The four study groups were well matched, and the results are highly significant, however they will have to be evaluated in a larger trial.

Our findings cannot be extrapolated as proof that ICU patients receiving 1 mg/kg QD are better protected than those receiving a standard dose of 40 mg QD, as anti-Xa activity is only a surrogate parameter. This could only be proven with adequately powered clinical trials. It remains to be established whether these results obtained with enoxaparin can be generalised to other LMWHs.

## Conclusions

Our study illustrates the inadequacy of the current standard doses of enoxaparin 40 mg QD (Europe) or 30 mg BID (North America) to yield target therapeutic range anti-Xa in critically ill patients weighing 50 to 90 kg.

It also demonstrates a clear relationship between anti-Xa levels and the dose of enoxaparin, with higher doses yielding better peak anti-Xa levels. Higher doses also allowed the establishment of near steady-state levels from the first day of enoxaparin administration.

The dual risks of VTE and bleeding, coupled with an overwhelming tendency to exclude patients with AKI continues to complicate the establishment of a strategy for thromboprophylaxis in this patient population. A weight-based dose yielded the best anti-Xa levels without an accompanying risk of bioaccumulation, and may thus be more appropriate, convenient and effective. A new study using 1 mg/kg QD enoxaparin has recently been approved by the Danish Ministry of Health. It will be conducted in AKI patients upon commencing continuous veno-venous haemofiltration, and will have clinical endpoints such as the occurrence of VTE and bleeding, length of stay, number of ventilator-free days, and mortality.

## Key messages

• doses of 40 mg QD and 30 mg BID enoxaparin yield sub-therapeutic levels of anti-Xa in critically ill patients.

• a dose of 1 mg/kg QD enoxaparin is more likely to achieve a target anti-Xa range of 0.1 to 0.4 U/ml.

• thromboprophylactic studies with clinical endpoints should be vigorously pursued.

## Abbreviations

AKI: acute kidney injury; anti-Xa: anti-factor Xa; APACHE II: Acute Physiology and Chronic Health Evaluation; aPTT: activated partial thromboplastin time; AT: antithrombin; BID: twice daily; COPD: chronic obstructive pulmonary disease; CrCl: creatinine clearance; GCP: Good Clinical Practice; ICU: intensive care unit; INR: international normalised ratio; LMWH: low- molecular-weight heparin; N: number; QD: once daily; SAPS II: Simplified Acute Physiology Score II; sc: subcutaneous; SD: standard deviation; SOFA: Sequential Organ Failure Assessment score; VTE: venous thromboembolism.

## Competing interests

The authors declare that they have no competing interests.

## Authors' contributions

PT conceived the research, revised the manuscript, and secured funding for the project. SR designed the study, enrolled and followed up patients, interpreted the results, performed the statistical analysis, and drafted the manuscript. AZ and ULL enrolled and followed up patients and revised the manuscript. CE assisted with the statistical analysis and revised the manuscript. MN assisted with the interpretation of the results and revised the manuscript. BR carried out the chromogenic assays and revised the manuscript. All authors read and approved the final manuscript.
